# Towards Real-Time Aquatic Monitoring of Strontium-90: Performance Evaluation of CaF_2_(Eu) and ZnSe(Al,O) Scintillators

**DOI:** 10.3390/s26030900

**Published:** 2026-01-29

**Authors:** Arjana Kolnikaj, Kelum A. A. Gamage, Olaoluwa Popoola, James Graham, Antonio Di Buono

**Affiliations:** 1 James Watt School of Engineering, University of Glasgow, Glasgow G12 8QQ, UK; arjana.kolnikaj@glasgow.ac.uk (A.K.);; 2 United Kingdom National Nuclear Laboratory, Havelock Road, Workington CA14 3YQ, UK

**Keywords:** beta radiation detection, silicon photo multipliers, europium-doped calcium fluoride, aluminium oxygen-doped zinc selenite, inorganic scintillators, Sr-90 detection, nuclear decommissioning

## Abstract

A compact, in situ beta-spectroscopy approach for real-time monitoring of Strontium-90 (Sr-90) in contaminated groundwater has been investigated. Two inorganic scintillators, CaF_2_(Eu) and ZnSe(Al,O), were coupled to silicon photomultipliers (SiPMs) and evaluated experimentally using custom front-end electronics. This was also modelled with Monte Carlo simulations using the Geant4 toolkit. Although simulations correctly predicted ZnSe(Al,O) has an advantage due to its higher light yield and optical transport, experimental measurements additionally revealed practical limitations of the readout electronics which were not captured in the simulation model. ZnSe(Al,O) showed excellent agreement with the simulated detector response (*R*^2^ ≈ 0.86; $\chi^{2}$/NDF ≈ 27). It also attains a higher relative detection efficiency (∼61.5%), yielding faithful capture of the composite Sr-90/Y-90 spectrum with only minor suppression at the extreme high-energy tail. CaF_2_(Eu) exhibits a deficit at low-mid energies and an apparent enhancement in the high-energy tail. This is consistent with threshold and photon-statistics losses and leads to poorer agreement with simulation ($\chi^{2}$/NDF ≈ 179) and lower overall efficiency (∼22.7%). These findings identify ZnSe(Al,O) as the stronger candidate for an underwater, in situ Sr-90 beta-spectroscopy system and motivate targeted optimisation of SiPM coupling and crystal-edge reflectivity in future designs.

## 1. Introduction

Part of the UK’s nuclear decommissioning efforts involve monitoring the presence of Sr-90 in the environment. At sites such as Sellafield, historical operations have resulted in the contamination of soils and underlying aquifers. Total beta screening shows many groundwater samples exceed the WHO drinking water guidelines of 1 Bq L^−1^, with the highest 2016 annual average of 105,553 Bq L^−1^ measured in monitoring wells within the separation area [1]. Groundwater monitoring is needed to protect the workforce, the public, and the environment, and it is a statutory requirement under the UK Environmental Permitting regulations. It is also critical for characterising the distribution, movement and temporal evolution of radionuclides within the complex aquifer systems. Sr-90 is a radionuclide of interest due to its solubility and high-mobility in water. Current groundwater monitoring procedures on nuclear sites involves the manual sampling of groundwater from boreholes using a number of recognised approaches [2]. Samples acquired during these procedures are then transported to a laboratory for analysis. Typical analytical techniques include radiochemical separation, liquid scintillation counting (LSC), Cherenkov counting [3], extraction chromatography [4], and precipitation [5]. This report presents progress towards an alternative, non-destructive, in situ method for monitoring Sr-90 directly within groundwater. At present, there are no commercially available products capable of performing real-time beta spectroscopy in underwater environments. Commercial beta radiation detectors such as Mirion’s airborne alpha/beta particulate monitor [6], Thermo Fisher’s RadEye [7] or Kromek’s alpha/beta probe [8] offer counts-based discrimination rather than spectral beta-energy resolution. They are also not capable of resolving beta energy spectra in water. Field-deployable spectrometers from major manufacturers are almost exclusively gamma-ray systems. Academic work on beta detection has likewise been limited to laboratory-based prototypes including plastic scintillators, Cherenkov detectors, and semiconductor-based detectors [9,10,11,12]. None of these prototypes are capable of real-time operation in groundwater, and there are no deployable solutions for distinguishing Sr-90 directly within groundwater. Consequently, a clear technological gap exists in both research and industry. The work presented here aims to address this gap through the development and experimental validation of a compact, scintillator–SiPM-based beta detection system designed for aquatic deployment.

For monitoring Sr-90 in groundwater, two inorganic scintillators: CaF_2_(Eu) and ZnSe(Al,O), were selected for evaluation. Both materials possess relatively low density which reduces their gamma sensitivity. They are both non-hygroscopic, making them suitable for aquatic environments, and they have relatively high light yields. Their emission spectra are also well matched to the photon detection range of silicon photomultipliers (SiPMs). CaF_2_(Eu) serves as a benchmark material due to its extensive use in radiation detection, whereas ZnSe(Al,O) was identified as a promising alternative with favourable optical and scintillation characteristics that have received comparatively little experimental attention. On this basis, ZnSe(Al,O) was hypothesised to be the more effective candidate for compact, in situ beta spectroscopy.

The detectors developed in this work integrate these scintillators with SiPM readout. SiPMs are increasingly being adopted in radiation detection due to their compactness, robustness, and low operating voltage. They provide photomultiplier-like gain (on the order of 10^6^), high photon detection efficiency, and insensitivity to magnetic fields [13]. These properties make them an ideal choice for a portable, field-deployable spectroscopic system.

## 2. Materials and Methods

The current configurations being explored are Hamamatsu’s (Hamamatsu Photonics, Hamamatsu City, Shizuoka Pref., 430-8587, Japan) S13360 3075-PE SiPM (s13360) c, which is paired to CaF_2_(Eu), and Hamamatsu’s S14420 3050-MG SiPM (S14420) to a ZnSe(Al,O) (both crystals were sourced from Advatech, Woodford Green, IG8 9HB, UK). Table 1 and Table 2 summarise the specifications of the sensor components used.

**Figure 1:**
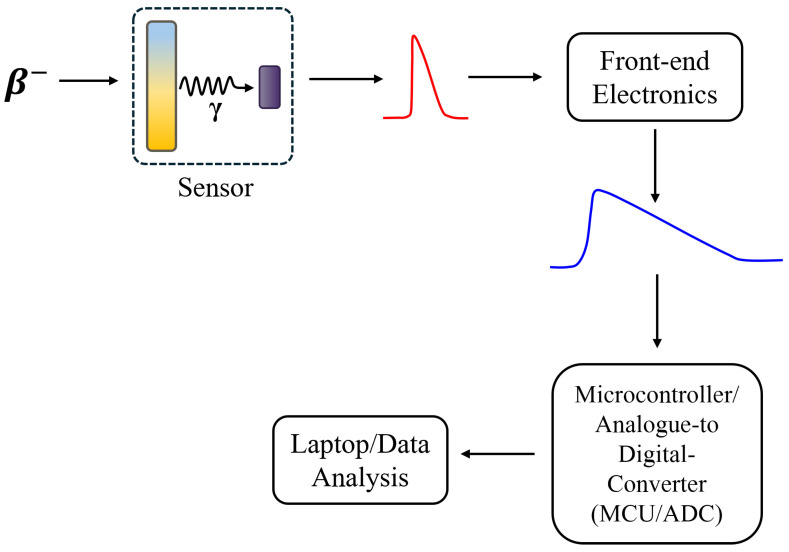
Block diagram of the system. The sensor comprised of the 3D-printed case housing the crystal and SiPM. This connects to front-end electronics and an Arduino Giga MCU, which digitises the SiPM signals. The current pulse produced by the sensor is amplified and held long enough for the MCU to sample the amplitude of the peak. Peak amplitudes are then transmitted to a python notebook which was used for data analysis.

Figure 1 depicts a block diagram of the detector system. Ionising radiation is incident on the sensor, where it is converted into photons by the scintillator and these photons are then collected by the SiPM. The SiPM produces a measurable current pulse that is indirectly related to the energy deposited in the scintillator. Custom readout electronics amplify and shape this signal so that it can be digitised by a microcontroller’s ADC (Arduino Giga, Arduino SA, Corso San Gottardo 6A CH-6830 Chiasso, Switzerland). The SiPM is biased above its breakdown voltage using a CAEN A7585DU SiPM power module (Viareggio, Italy). The signals produced by the SiPM are on the order of a 10s to 100s of mV. They are fed into a high speed op-amp with a gain set to 300. After amplification, a peak-hold stage captures the pulse’s maximum voltage and stretches the signal in time so that it can be sampled. This stage consists of a 1 nF capacitor to temporarily hold the peak and a 22 kΩ resistor to set the discharge (fall) time constant. A 300 Ω resistor is placed at the output of the amplifier to isolate it from the peak-hold capacitor; this prevents the capacitor from directly shorting or overloading the amplifier output when it charges. The amplifier and peak-hold circuits were powered by two 9 V batteries. The ADC assigns discrete digital values to the incoming analogue signals, capturing their peak amplitude and allows the microcontroller (MCU) to store or transmit this data. The data is then subsequently processed in a computer. A list of all software used during this entire investigation is shown in Table 3.

### 2.1. Simulation Model

**Figure 2:**
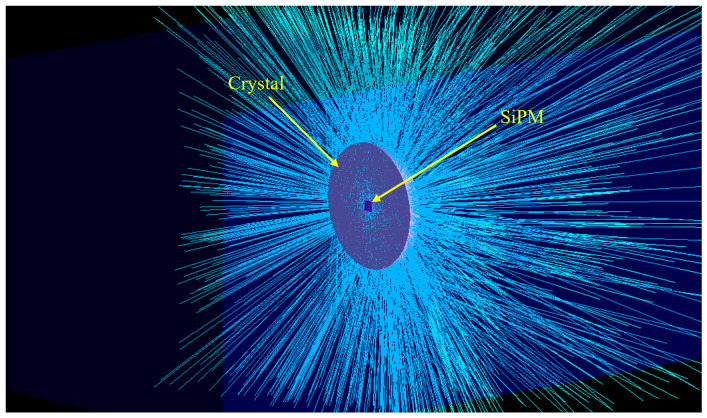
3D render from Geant-4 simulation showing detector in envelope (air) which can be set to match environment detector is in. The SiPM can be seen in pink attached to the scintillation crystal in white. Cyan tracks are optical photons.

The detector geometry in Geant4 was modelled as a cylindrical scintillator (CaF_2_(Eu) or ZnSe(Al,O)) positioned within an air-filled world volume, where the term ‘world volume’ refers to the outermost simulation volume. The crystal dimensions and material properties including: density, refractive index, scintillation yield, and decay constants were defined according to experimental specifications. The SiPM was modelled as a 3 × 3 mm^2^, 0.5 mm thick, rectangular, ideal photon-catching surface, located directly behind the crystal at the SiPM plane. Figure 2 visualises the simulation.The detector’s properties, such as the photon detection efficiency and gain were applied during post-processing when converting detected photons into the simulated electrical signal. 1 × 10^5^ Sr-90 decays were simulated and their passages tracked through the detector, recording energy deposited in the crystal. The scintillation light produced by the beta particles interacting with the crystal medium were tracked using ray-tracing and any photons incident on the SiPM were recorded.

### 2.2. Experimental Method

**Figure 3:**
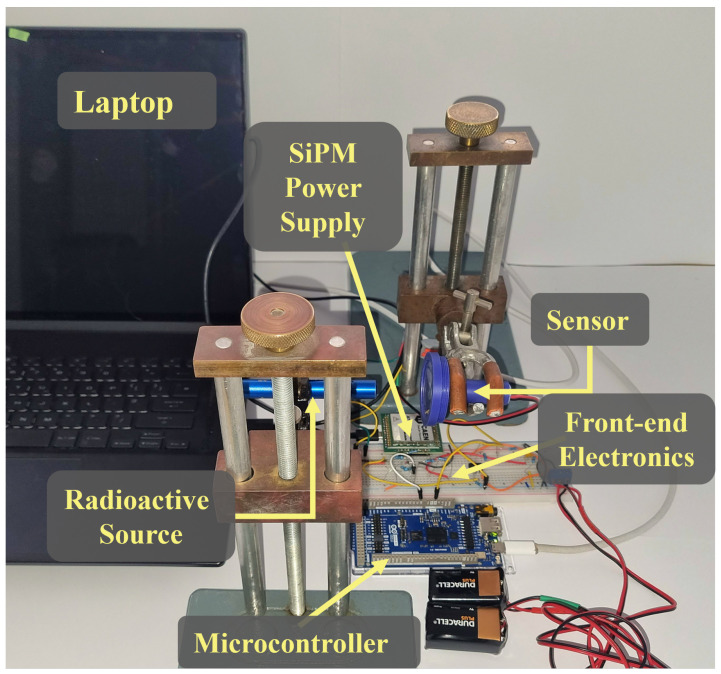
Experimental setup for Sr-90 detector testing. The SiPM–scintillator sensor connects to custom front-end electronics and an Arduino Giga for data acquisition, with bias supplied by an external power module. The radioactive source is mounted in front of the detector using an adjustable holder to maintain consistent geometry during measurements.

The experimental setup shown in Figure 3 consisted of a 3D-printed detector housing containing the scintillator and SiPM assembly, with the Sr-90 source placed directly against the sensor face to maximise beta interaction probability. The scintillator crystal was wrapped in PTFE tape to enhance internal light reflection and minimise photon loss through the crystal edges [18]. Both the source and detector were held securely in alignment using clamp stands. The sensor assembly was connected to a breadboard containing the front-end readout electronics, bias supplies (a CAEN A7585DU high-voltage module for the SiPM and two 9 V batteries for the circuit), and an Arduino Giga MCU for digitisation and data acquisition. The MCU sampled the peak-held analogue signal using its on-board ADC and streamed the digitised pulse-height data to a computer for storage and analysis. Background spectra were recorded before each measurement and subtracted from the corresponding source spectra. A Sr-90 source with an activity of 2.75 × 10^3^ Bq was used (activity data supplied by the University of Glasgow physics department), and approximately 5000 pulses were collected per detector at optimised trigger thresholds.

To further assess the readout performance, a threshold-scan analysis was conducted, in which the trigger level was incrementally varied and the mean pulse height at each setting was recorded for 5000 pulses. The resulting threshold–pulse-height trends were evaluated to study the stability and linearity of the system. In future iterations, a time-based acquisition approach will be implemented to quantify detection rate as a function of threshold.

## 3. Results

### 3.1. Energy Calibration

Traditional energy calibration methods rely on discrete gamma-ray peaks, which are not suitable for this detector system, this is because it has been optimised for beta radiation detection. To enable a quantitative energy reconstruction from the data recorded, a theoretical relationship has been derived to link the energy deposited in the detector (scintillator-SiPM assembly) to the output voltage response of the readout electronics. Integrated charge and energy deposited in a detector are directly proportional. In order to calculate the charge produced by a scintillation counter, the number of photons must be calculated. When a beta particle deposits its energy, E in the scintillator, the atoms in the crystal are excited and produce visible photons proportional to the deposited energy. The proportionality constant is the scintillator’s light yield, $\epsilon_{l}$ (photons per MeV). For a deposited energy, *E* the number of scintillation photons $N_{Scint}$ is defined as [19]:

(1) $$N_{scint}=E\times \epsilon_{l}$$

Only a fraction of the photons reach the SiPM and trigger avalanches. The probability of an incident photon being converted into an electrical signal is defined as the photon detection efficiency (PDE) and depends on the quantum efficiency (ratio between detected photons and incident photons striking the sensitive area of the SiPM), triggering probability and fill factor [20]. The number of photoelectrons, $N_{PE}$, which are the electrons produced as a result of photons striking the SiPM, is defined by

(2) $$N_{PE}=N_{Scint}\times PDE$$

Each detected photon sets off an avalanche of charge carriers, $N_{e}$ which depend on the gain of the SiPM, *G* such that [21]

(3) $$N_{e}=N_{PE}\times G$$

Equation (3) can trivially be converted to charge, *Q* in SI units by multiplication with electron charge, *e*, yielding

(4) $$Q=eN_{e}$$

*Q* can also be written as

(5) $$\bar{Q}=It$$

In the front-end electronics of the detector, the current produced by the SiPM passes through a load resistor with resistance, *R* and lasts for a characteristic pulse duration, *t*. Using Ohm’s law, we obtain

(6) $$\bar{Q}=\frac{V}{R}\cdot t$$

Using Equations (4) and (6) and substituting in Equation (3) and using Equation (2);

(7) $$\begin{array}{c} \frac{V}{R}\cdot t=eN_{e} \end{array}$$

(8) $$\begin{array}{c} \frac{V}{R}\cdot t=eG\cdot PDE\cdot N_{Scint} \end{array}$$

And using Equation (1) and rearranging for voltage:

(9) $$\bar{V}=\frac{e\cdot G\cdot R\cdot PDE\cdot E\cdot \epsilon_{l}}{t}$$

Equation (9) gives the relationship between voltage and energy deposited in the scintillator where the physical constants are defined by the scintillator-SiPM assembly and electronics parameters.

**Figure 4:**
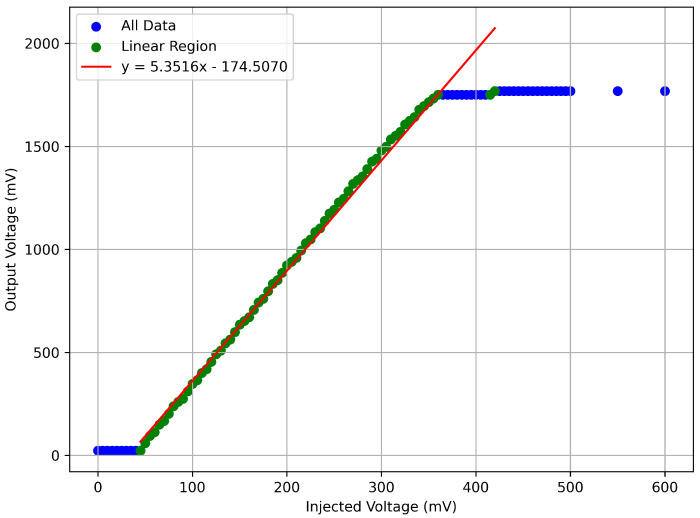
Plot showing the injected voltage vs the output from the readout circuit. Known voltages were injected into the circuit and a multimeter was used to read the output voltage after amplification.

Figure 4 shows the relationship between the injected voltage and the output voltage from the readout. The readout circuit exhibits a strong linear amplification up to ∼350 mV (injected). Beyond this point, saturation occurs after ∼1.8 V (output). Linearity confirms consistent gain in this region which validates the circuit’s suitability for pulse-height preservation.

**Figure 5:**
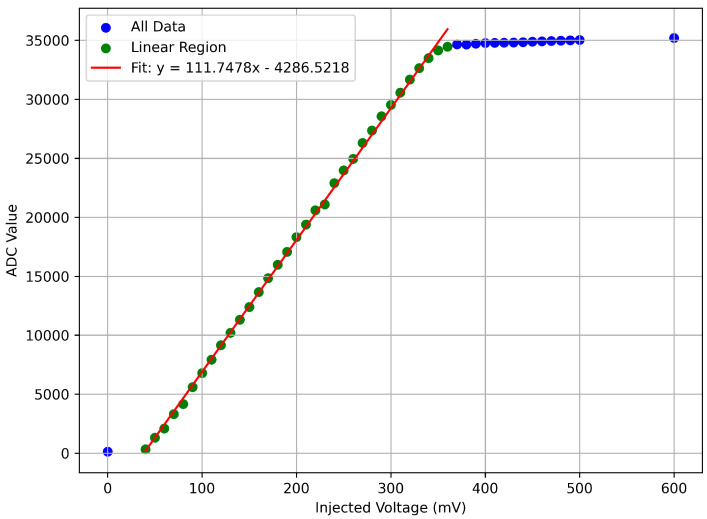
Voltage was steadily increased and the raw ADC number recorded by the MCU. The linear region (green) and fit (red) were used to establish the ADC-to-voltage conversion.

The ADC in the MCU was then calibrated to record pulse amplitudes (ADC values) as voltages. A linear response in Figure 5 is observed between ∼50–380 mV. Saturation occurs around ∼400 mV which is in agreement with the linearity measurement between input and output voltages (Figure 4). The pulse amplitudes generated by Sr-90 beta interactions fall within the linear range. However, it is important to note that purpose of this analysis was to verify the linearity of the electronics and microcontroller ADC and to establish a conversion between the two. Once data was recorded, voltages taken from the MCU were converted into energy using Equation (9).

### 3.2. Simulations Comparing Efficiency

**Figure 6:**
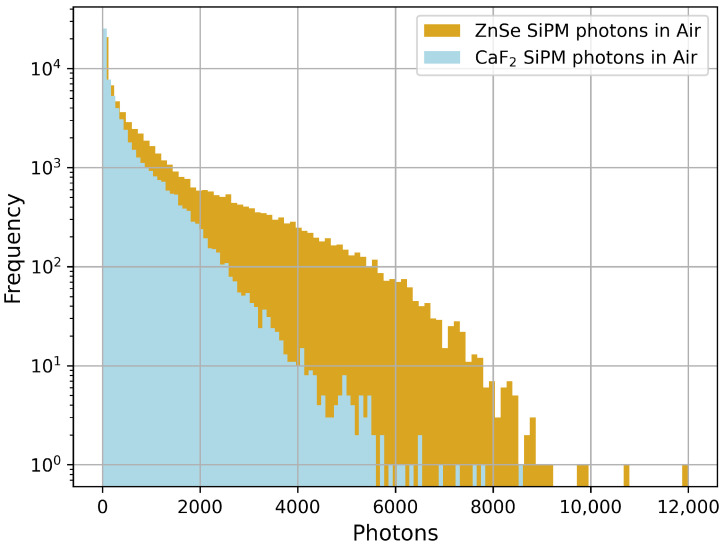
Simulated photon distributions at the SiPM surface for ZnSe(Al,O) (gold) and CaF_2_(Eu) (blue) exposed to Sr-90. Each histogram shows the number of scintillation photons per event incident on the SiPM active area. The ZnSe(Al,O) detector demonstrates a higher and broader photon yield, indicating improved light output and light collection.

Figure 6 shows the simulated distributions of scintillation photons reaching the SiPM surface for both ZnSe(Al,O) and CaF_2_(Eu) under Sr-90 excitation. The histograms were obtained by recording the number of photons incident on the SiPM active area per event in a Geant4 simulation. The ZnSe(Al,O) distribution extends to higher photon counts and displays a broader shape, indicating that more scintillation photons are produced and successfully transported to the detector face. In contrast, CaF_2_(Eu) shows a narrower distribution concentrated at lower photon counts, consistent with its lower light yield and refractive index.

**Figure 7:**
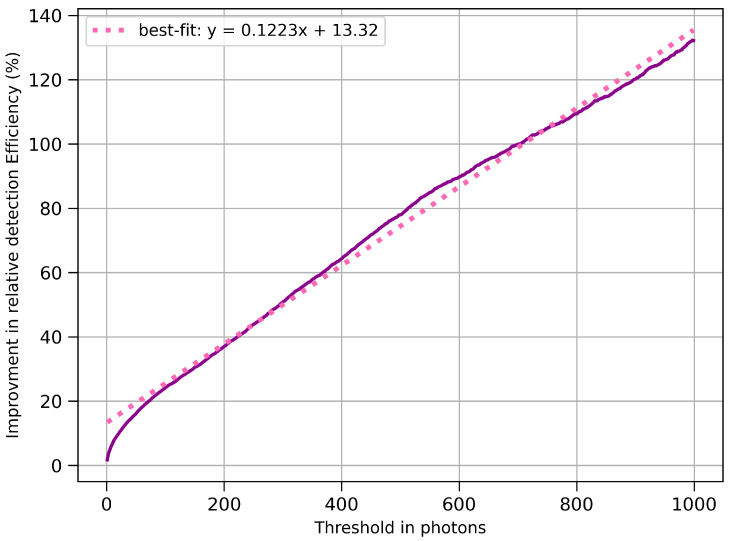
Simulated relative detectionefficiency improvement of ZnSe(Al,O) over CaF_2_(Eu) as a function of photon threshold. The linear fit indicates an approximate 12% increase in efficiency per 100 additional photons.

Figure 7 presents the simulated relative detection-efficiency improvement of ZnSe(Al,O) over CaF_2_(Eu) as a function of photon-count threshold. For each threshold level, the number of events exceeding the threshold was computed for both materials, and the relative improvement was evaluated. The plot reveals a near-linear trend, with ZnSe(Al,O) maintaining a consistent advantage at all thresholds. The linear regression indicates an approximate 12% gain in detection efficiency for every additional 100 photons.

### 3.3. Comparison of Experimental Data with Simulation Data

**Figure 8:**
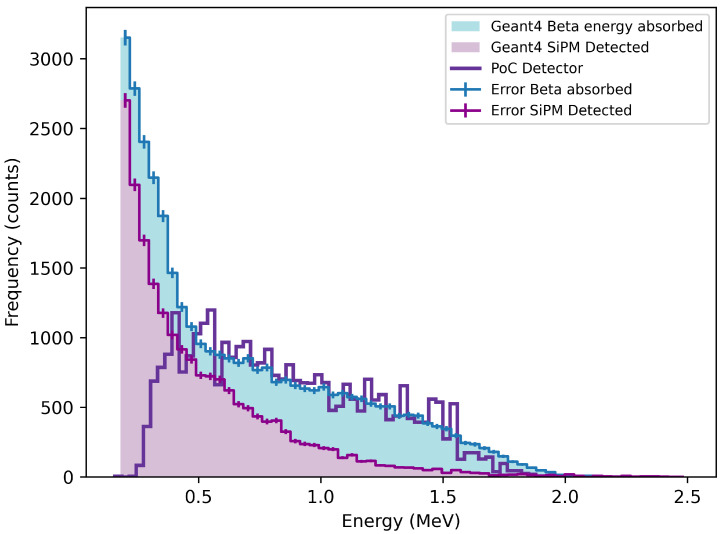
Calibrated energy spectrum of Sr-90 measured with the CaF_2_(Eu) proof-of-concept (PoC) detector, shown alongside the corresponding Geant4-simulated Sr-90 spectrum and simulated detector response.

**Figure 9:**
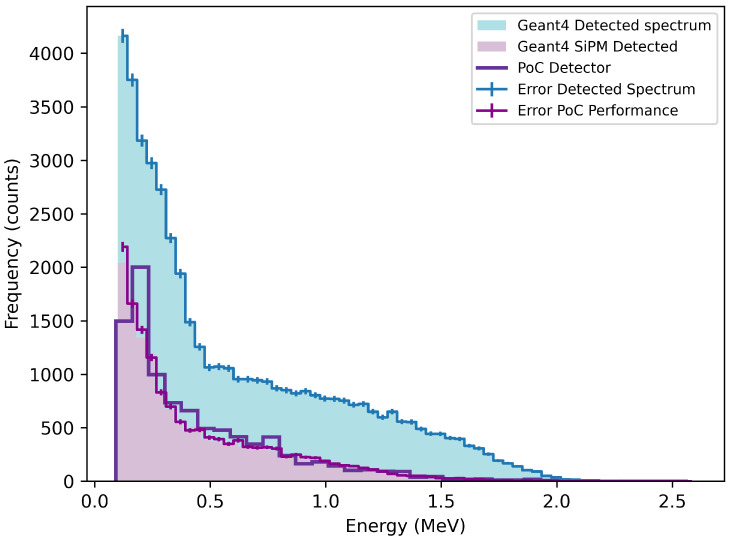
Calibrated energy spectrum of Sr-90 measured with the ZnSe(Al,O) detector, compared with the corresponding Geant4-simulated Sr-90 spectrum and simulated detector response.

Figure 8 and Figure 9 compare the experimental spectra obtained using a Sr-90 source with corresponding Geant4 simulations. The simulations show the energy deposited in the scintillator and the corresponding signal detected by the SiPM. The experimental spectrum, recorded using the setup in Figure 3, is shown with background subtracted. The shaded regions also have error bars that represent the statistical uncertainty on the simulated spectra arising from finite Monte Carlo statistics, and do not indicate a discrepancy between simulation and experimental data. Figure 8 shows the measured Sr-90 spectrum from the CaF_2_(Eu) detector alongside the Geant4-simulated energy absorbed by the scintillator and the SiPM-detected signal. The experimental data appears broader but clearly extends beyond the simulated signal at higher energies. This tailing suggests the detector is responding to higher-energy betas from the Y-90 daughter, consistent with the expected decay endpoint of 2.28 MeV. A reproducible local enhancement is observed near 0.6 MeV, close to the most probable beta energy of Y-90. While the nature of beta radiation does not permit precise spectroscopic peak identification, the persistence of this feature under variations in binning and its agreement with the simulated Sr-90/Y-90 response indicate sensitivity of CaF_2_(Eu) to the Y-90 component of the decay chain.

In comparison, Figure 9 presents the equivalent spectrum for the ZnSe(Al,O) detector. The experimental result closely follows the shape and extent of the SiPM-detected simulation. The tail-off occurs near 1.5 MeV, and the spectrum peaks just below 0.2 MeV, which is in line with Sr-90’s most probable energy. This suggests ZnSe(Al,O) is more selective for the parent Sr-90 decay and does not significantly respond to the higher, energy Y-90 beta component.

Table 4 summarises the key findings from Figure 8 and Figure 9. The efficiency was calculated as the ratio between the experimentally observed count rate and the simulated SiPM-detected event rate for each material independently. This definition differs from the relative efficiency trend shown in Figure 7, which represents the incremental improvement in detection efficiency between ZnSe(Al,O) and CaF_2_(Eu) as a function of photon-count threshold, rather than an absolute experimental-to-simulation efficiency. The two quantities therefore describe different aspects of detector performance and are not directly comparable. However, both metrics are significant and, taken together, independently indicate superior performance of ZnSe(Al,O).

### 3.4. Effect of Trigger Threshold on Pulse Height Distribution

**Figure 10:**
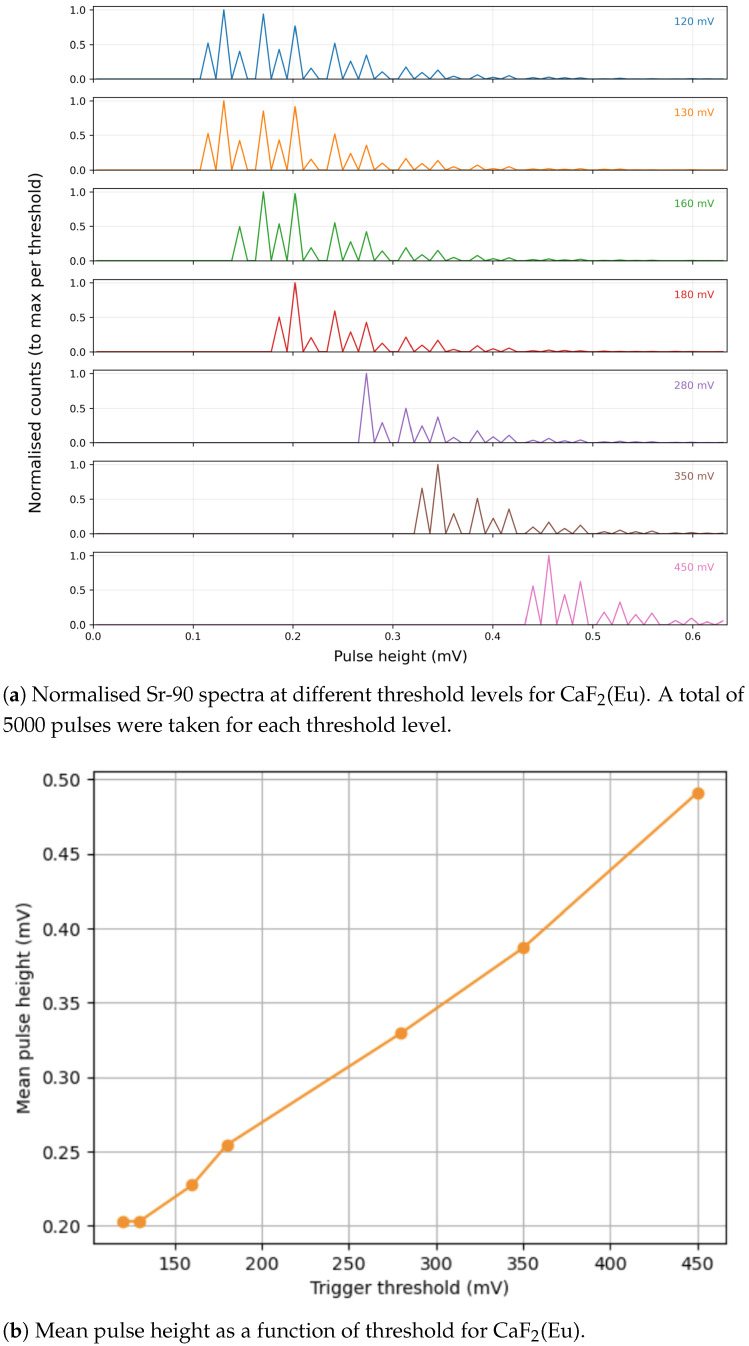
Experimental results for the CaF_2_(Eu) detector showing (**a**) threshold-dependent spectral behaviour and (**b**) variation of mean pulse height with threshold.

**Figure 11:**
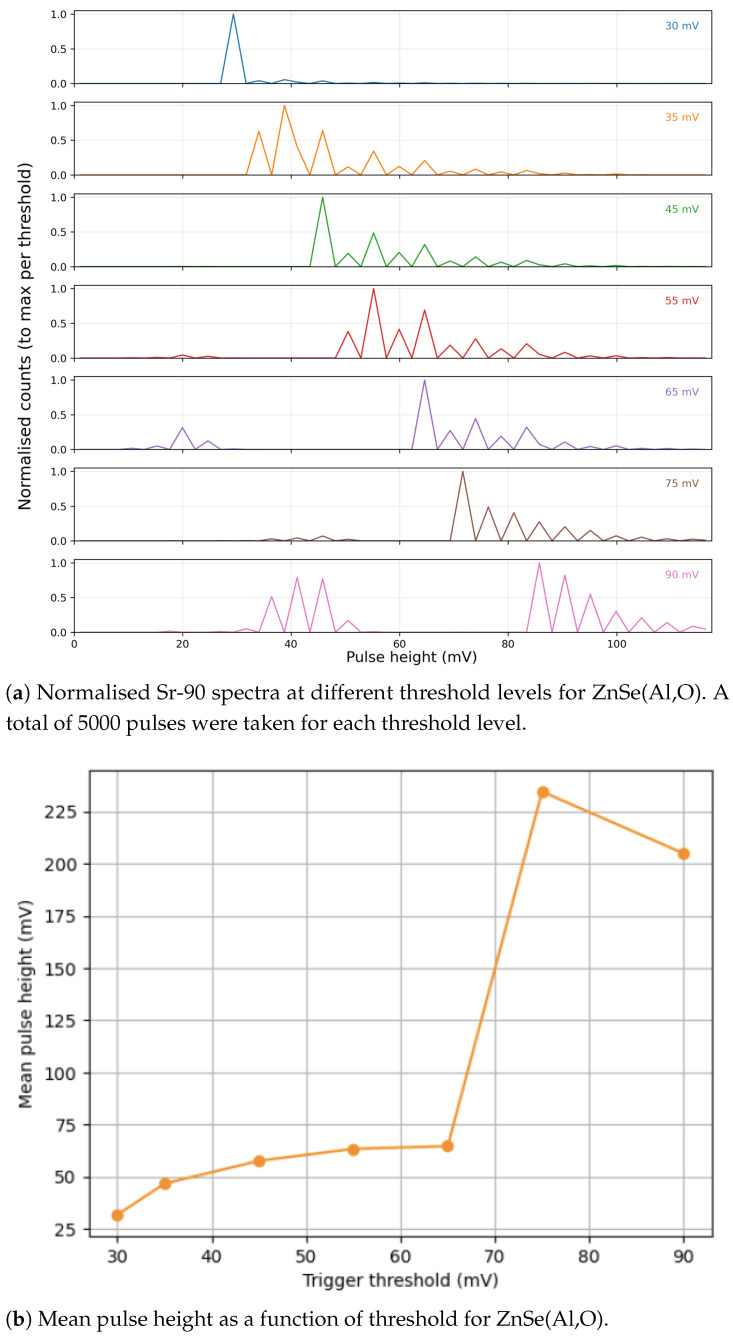
Experimental results for the ZnSe(Al,O) detector showing (**a**) threshold-dependent spectral behaviour and (**b**) variation of mean pulse height with threshold.

The threshold level for triggering using leading edge discrimination was investigated by analysing the signal seen from a Sr-90 source with varying threshold levels. The results of this can be seen in Figure 10 and Figure 11. Table 5 provides the optimal thresholds for each detector and a summary of their behaviour. It can be seen from Figure 10b that the mean pulse height initially did not increase but then varied linearly with threshold voltage across the range tested, showing a smooth response (to varying threshold) and indicating a linear response with increasing pulse height across the voltage range that the detector outputs. It can also be seen from Figure 10a that the leading edge discriminator is cutting the spectrum as expected, rejecting lower pulse heights without distortion to the rest of the spectrum. Figure 10b is also of use in determining the optimum threshold value, as it is likely that most of the baseline noise has been rejected by 150 mV, and by 200 mV, the signal is clearly in a fairly linear region.

ZnSe(Al,O) exhibits a less linear trend than CaF_2_(Eu), as seen in Figure 11. A smooth, gently curved response is observed at low amplitudes, followed by a sharper increase in mean voltage in Figure 11b. This behaviour is consistent with the longer scintillation decay profile of ZnSe(Al,O) and the way its pulse shape interacts with the readout electronics. It indicates where tailoring the shaping parameters can further improve linearity in future iterations. At present, the results remain stable, reproducible, and confirm ZnSe’s compatibility with SiPM-based readout.

## 4. Discussion

### 4.1. Simulations Comparing Efficiency of ZnSe(Al,O) and CaF_2_(Eu)

The plots shown in Section 3.2 indicate that ZnSe(Al,O) is more efficient than CaF_2_(Eu) in producing and transmitting scintillation photons to the SiPM. Its broader photon distribution and sustained response at higher thresholds suggest that ZnSe(Al,O) can detect a greater range of beta-induced events. In contrast, CaF_2_(Eu) shows a rapid drop in detected photons beyond higher thresholds, consistent with its lower light yield. Overall, the simulated behaviour suggests ZnSe(Al,O) would deliver improved signal-to-noise ratio and its higher light-yield would be useful for detecting lower-energy beta events.

### 4.2. Comparison Between Simulation and Experimental Data for Both Detectors

A previous study used CaF_2_(Eu) with an SiPM from the S13360 series to detect Sr-90 in water for environmental monitoring [23]. While the goal was similar, there are key differences. Their setup used an off-the-shelf amplifier and a lab-based MCA, and their in-water test lasted just 400 s with few events recorded above 20 keV. They reported a detection depth of 7 mm in water but had a polycarbonate window in front of the crystal. This would have attenuated beta particles and was also not needed since CaF_2_(Eu) is non-hygroscopic. Additionally, the authors noted their electronics did not work reliably and had to be excluded—which is why their system relied on a lab based multichannel analyser. They also noted signal degradation after 10 m of cable, which would limit deployment.

Since ZnSe(Al,O) is an unexplored material, particularly for beta spectroscopy, there are not many relevant studies to compare with the work carried out here. Galkin et al. conducted a study in 2021 evaluating the performance of ZnSe(Te) (or Al doped) under alpha excitation at low temperatures [24]. Their study showed that ZnSe(Al) exhibits higher light yield and faster decay kinetics than ZnSe(Te), especially below 100 K. Although application and operational temperature differ from the study here, the high photon yield observed for ZnSe(Al) align with the general performance advantages observed here for ZnSe(Al,O). Another earlier study investigated beta detection using a thin layer of ZnSe(Te) grains dispersed on a wedge-shaped light guide, conducted by Gavrylyuk et al. [25]. The Sr-90 spectrum produced bears strong resemblance with the spectrum seen in Figure 9. Their study demonstrated the feasibility of using ZnSe-based scintillators for beta spectroscopy with a focus on maximising surface area. In contrast, the ZnSe(Al,O) detector built here is based around a single crystal with direct SiPM coupling. It is able to capture a Sr-90 spectrum and avoids the need for complex optical transducers.

### 4.3. Trigger Threshold and Pulse Height Distribution

Further study is being undertaken to study the full pulse profile generated from each material, and the observed characteristics will be used to improve the readout circuit and tailor it to get the best from each material. Further tests will also be conducted to examine the rate capabilities of each detector, as well as methods for pileup rejection. Figure 10 and Figure 11 show that the pulse conditioning and readout are currently more mature for the CaF_2_(Eu)-based detector. However, ZnSe(Al,O) demonstrates strong potential, with further optimisation of readout electronics, represents a highly suitable candidate for this application.

## 5. Future Work

The results presented here represent a proof-of-concept evaluation performed under controlled laboratory conditions in air. Future work will therefore focus on validating detector performance under conditions representative of in situ groundwater monitoring and on progressing towards field deployment. Laboratory measurements will be conducted with the detector held in direct contact with contaminated groundwater samples from Sellafield. In this instance, radionuclide characterisation data would be made available by Sellafield from their current, established monitoring techniques. This will allow the direct comparison between the measured beta spectra with the detector system and known isotopic compositions.

In parallel, the detector design will be further optimised by implementing an SiPM array (as opposed to the single SiPM configuration currently) to improve light-collection efficiency, absolute detection efficiency, and spectroscopic performance. Mechanical development will focus on a waterproof detector housing suitable for borehole deployment, while maintaining direct optical contact between the scintillator assembly and the surrounding water to minimise additional beta attenuation. Successful laboratory validation in water will form the basis for subsequent deployment trials in an operational borehole environment, representing the final stage of the system’s development towards real-time, in situ groundwater monitoring.

## 6. Conclusions

This work assessed CaF_2_(Eu) and ZnSe(Al,O) for use in a compact, in situ beta-spectroscopy system for monitoring Sr-90 in groundwater. The process involved characterising the linearity and dynamic range of the front-end electronics and ADC, calibrating pulse-height response, modelling photon production and transport using Geant4, and directly comparing background-subtracted experimental spectra against simulated detector responses under identical source conditions. The results showed that ZnSe(Al,O) offers stronger overall performance for this application. ZnSe(Al,O) achieved a relative detection efficiency of 61.5% compared to 22.7% for CaF_2_(Eu), and demonstrated substantially improved spectral agreement with simulation ($\chi^{2}$/NDF = 27 versus 179). Its higher light yield and refractive index led to higher relative efficiency and more consistent detection of higher-photon events, which in turn produced experimental spectra that aligned well with the simulated detector response. CaF_2_(Eu) also produced a usable Sr-90/Y-90 spectrum, but its lower photon statistics and higher effective threshold resulted in reduced sensitivity at low and mid energies. The threshold-scan studies confirmed that both detectors can be operated with stable discrimination, with ZnSe(Al,O) requiring further optimisation of the readout to accommodate its longer decay time. Taken together, the findings indicate that ZnSe(Al,O) is the more suitable material for a field-deployable aquatic beta spectrometer, while CaF_2_(Eu) remains a viable lower-cost alternative.

## Figures and Tables

**Table 1:** Summary of scintillator characteristics [14,15].

	CaF_2_(Eu)	ZnSe(Al,O)
Density (g cm^−3^)	3.18	5.27
Refractive index	1.47	2.41
Light yield (photons keV^−1^)	19	50
Emission wavelength (nm)	435	605
Diameter & thickness (mm)	38, 3.6	42, 2.1
Decay Time (μs)	0.95	25

**Table 2:** Summary of SiPM specifications [16,17].

SiPM Model No.	S13360-3075PE	S14420-3050MG
Peak sensitivity wavelength (nm)	450	600
Refractive index of window	1.55	1.49
Photosensitive Area (mm)	3.0 × 2.0	ϕ3.0
Photo detection efficiency (PDE) (%)	50	40
Gain	1.7 $\times \;10^{6}$	3.6$\;\times \;10^{6}$
Breakdown voltage (V)	53 ± 5	42 ± 5

**Table 3:** List of software used and what it was used for.

Software Name	Use
Python, v7.2.2	data analysis
ROOT, v6.37.01	data analysis
Geant4, v11.2.0	creating simulations
Arduino IDE, v2.3.5	programming the microcontroller
Zeus, v2022.05.12.0	controlling the SiPM power supply

**Table 4:** Comparison of detector performance metrics for CaF_2_(Eu) and ZnSe(Al,O).

Metric	CaF_2_(Eu)	ZnSe(Al,O)	Interpretation
Efficiency (%)	22.7	61.5	ZnSe ≈ 3× higher
$\chi^{2}$/NDF	179	27	ZnSe ∼7× better fit with simulation than CaF_2_(Eu)
R^2^ (shape)	–3.4	0.86	ZnSe matches spectrum
68% Width (MeV) [22]	0.85 (Exp)/0.57 (Sim)	0.61 (Exp)/0.64 (Sim)	ZnSe ≈ ideal
FWHM Resolution (%)	180	–	CaF_2_ very broad; ZnSe flat β tail

**Table 5:** Comparison of optimal trigger thresholds and observed behaviour for ZnSe(Al,O) and CaF_2_(Eu) detectors.

Detector	Approx. Optimal Threshold (mV)	Behaviour Summary
ZnSe(Al,O)	∼35–45	High signal yield, clean separation between noise and signal, and a stable plateau across the threshold range.
CaF_2_(Eu)	∼120–160	Weaker light output and narrower pulse distribution, with noise suppression achieved only at higher threshold values.

## Data Availability

Data of this research is available upon request via first author.
